# Cytoprotective activity of mitochondrial uncoupling protein‐2 in lung and spleen

**DOI:** 10.1002/2211-5463.12410

**Published:** 2018-03-12

**Authors:** Martin Jabůrek, Jan Ježek, Petr Ježek

**Affiliations:** ^1^ Department of Mitochondrial Physiology Institute of Physiology Academy of Sciences Prague Czech Republic

**Keywords:** antioxidative synergy, cytoprotection, mitochondrial phospholipase iPLA2γ, mitochondrial uncoupling protein UCP2, protein carbonylation

## Abstract

Mitochondrial uncoupling protein‐2 (UCP2) mediates free fatty acid (FA)‐dependent H^+^ translocation across the inner mitochondrial membrane (IMM), which leads to acceleration of respiration and suppression of mitochondrial superoxide formation. Redox‐activated mitochondrial phospholipase A2 (mt‐iPLA2γ) cleaves FAs from the IMM and has been shown to acts in synergy with UCP2. Here, we tested the mechanism of mt‐iPLA2γ‐dependent UCP2‐mediated antioxidant protection using lipopolysaccharide (LPS)‐induced pro‐inflammatory and pro‐oxidative responses and their acute influence on the overall oxidative stress reflected by protein carbonylation in murine lung and spleen mitochondria and tissue homogenates. We provided challenges either by blocking the mt‐iPLA_2_γ function by the selective inhibitor R‐bromoenol lactone (R‐BEL) or by removing UCP2 by genetic ablation. We found that the basal levels of protein carbonyls in lung and spleen tissues and isolated mitochondria were higher in UCP2‐knockout mice relative to the wild‐type (wt) controls. The administration of R‐BEL increased protein carbonyl levels in wt but not in UCP2‐knockout (UCP2‐KO) mice. LPS further increased the protein carbonyl levels in UCP2‐KO mice, which correlated with protein carbonyl levels determined in wt mice treated with R‐BEL. These results are consistent with the UCP2/mt‐iPLA_2_γ antioxidant mechanisms in these tissues and support the existence of UCP2‐synergic mt‐iPLA_2_γ‐dependent cytoprotective mechanism *in vivo*.

**Abbreviations**
BEL: bromoenol lactone
FA: fatty acid
FCCP: carbonyl cyanide *p*‐(trifluoromethoxy) phenylhydrazone
HEPES: 4‐(2‐hydroxyethyl)‐1‐piperazineethanesulfonate
IMM: inner mitochondrial membrane
LPS: lipopolysaccharide
MOPS: 3‐(*N*‐morpholino)propanesulfonate
mt‐iPLA_2_γ: mitochondrial calcium‐independent phospholipase A_2_, isoform γ
OMM: outer mitochondrial membrane
PUFA: polyunsaturated fatty acid
R‐BEL: 6E‐(bromoethylene)tetrahydro‐3r‐(1‐naphthalenyl)‐2H‐pyran‐2‐one
ROS: reactive oxygen species
TCA: trichloroacetic acid
UCP2: mitochondrial uncoupling protein‐2

The term ‘uncoupling’ of respiration refers to a H^+^ backflux across the inner mitochondrial membrane (IMM), leading to a dissipation of the protonmotive force (Δ*p*) established by the respiratory chain proton pumps 1, 2. Thus, a complete dissipation of Δ*p* short‐circuits the H^+^ current via the F_O_ subunit of ATP synthase and hence uncouples the respiration from the ATP synthesis.

The term ‘mild uncoupling’ was introduced to describe a partial dissipation of Δ*p*, resulting in a transient regulated H^+^ backflux across the IMM of much lower amplitude than the H^+^ flux through the F_O_ ATP synthase 2, 3. Mild uncoupling promotes higher rates of respiration and rates of H^+^ pumping and electron transfer (unless these are set on their maxima), which in turn attenuates superoxide formation rates at the sites of respiratory chain depending on Δ*p* (e.g., Complex I and III sites I_Q_ and III_Qo_) 3, 4, 5. In contrast, it can result in a slower electron transfer by the respiratory chain and slower proton pumping. As a result, such situation promotes a higher superoxide formation. The latter can be, however, employed by the cell as a signaling mechanism or, in contrast, mild uncoupling can attenuate the ongoing redox signaling 3, 4.

Mitochondrial uncoupling protein‐2 (UCP2) belongs to the SLC25 mitochondrial anion carrier gene family. UCP2 mediates fatty acid (FA)‐dependent H^+^ translocation across the IMM 6, 7, and pioneer reports 8, 9 identified UCP2 role in attenuation of mitochondrial formation of superoxide and downstream reactive oxygen species (ROS; reviewed in Refs 3, 4). Recently, we reported antioxidant activity by a synergy of UCP2 and the mitochondrial H_2_O_2_‐activated calcium‐independent phospholipase A2γ (mt‐iPLA2γ) 10, 11. iPLA2γ belongs to a group VI, also termed patatin‐like phospholipase domain‐containing lipases (PNPLAs), out of at least 30 different phospholipases A2 12, 13, 14, 15, 16, 17, 18, 19. The PNPLA specificity lies in the ability to cleave unsaturated FAs at the *sn*‐2 ester bond of membrane phospholipids, which includes oxidized aliphatic chains from cardiolipin 19, as well as the release of saturated FAs upon the *sn*‐1 ester bond cleavage 12, 13.

Our previous work is consistent with a redox activation of mt‐iPLA2γ leading to cleavage of FAs from the IMM and initiation of UCP2‐mediated protonophoretic function 10, 11, which results in a fine feedback downregulation of ROS production at increased mitochondrial ROS levels. Therefore, by cleaving FAs from IMM, the activated iPLA2γ initiates the UCP2‐mediated attenuation of superoxide formation, which subsequently contributes to the decrease in the *pro*‐oxidant redox state. Such repetitive cycles of mild uncoupling can significantly contribute to the overall redox homeostasis and cytoprotection. In addition, iPLA2γ promotes FA‐dependent mild uncoupling catalyzed by the adenine nucleotide translocase in isolated heart mitochondria 20, which can contribute to the antioxidant mechanism in tissues where UCP2 is absent. Thus, the UCP2 may provide a significant antioxidant protection in tissues or cell types where it is expressed, such as in lung, spleen, pancreas, and kidney, as well as in neuroglial, immune, or cancer cells (reviewed in Ref. 3).

In this work, we have tested the hypothesis of mt‐iPLA2γ‐dependent UCP2‐mediated antioxidant protection using lipopolysaccharide (LPS)‐induced *pro*‐inflammatory and *pro*‐oxidative responses in lung and spleen and their acute influence on the overall oxidative stress reflected by the protein carbonylation, which is an established marker of nonenzymatic, free radical‐mediated protein oxidation 21, 22. We have provided challenges either blocking the mt‐iPLA2γ function by a selective inhibitor R‐bromoenol lactone (R‐BEL) or ablating UCP2 gene (studying tissues of the UCP2‐knockout mice). We have found that LPS challenge increased the acute protein oxidation in lung and spleen to a similar extent in the UCP2‐KO mice and wt mice treated with R‐BEL, supporting the hypothesis that the antioxidant synergy of UCP2 and mt‐iPLA2γ belongs to significant cytoprotective mechanisms in these tissues.

## Materials and methods

### Animals

C57BL/6J wt and UCP2‐KO mice were obtained from the Jackson Laboratory (Bar Harbor, ME, USA). UCP2 ablation in the UCP2‐KO mice was confirmed by an inability of PCR to amplify *Ucp2* from genomic DNA and by western blotting (cf. fig. 6. in Ref. 10). Animals were bred and housed in certified housing according to the rules of the European Union and with approval of the Academy of Sciences of the Czech Republic and the Institute of Physiology licensing committee, which follows the Guide for the Care and Use of Laboratory Animals (1985), NIH, Bethesda, MD, USA, and the European Guidelines on Laboratory Animal Care.

### Treatments and tissue collection

Unless specified otherwise, all chemicals were from Sigma‐Aldrich (St. Louis, MO, USA, now Merck KGaA). For inducing pro‐oxidative conditions, both wt and UCP2‐KO mice were subjected to subcutaneous injection of R‐BEL (Cayman Chemical, Ann Arbor, MI, USA), 1 mg·kg^−1^ body weight, applied every 24 h three times (72 h total) together with intraperitoneal (i.p.) injection of sublethal dose of LPS s (from *Escherichia coli* O111:B4), 5 mg of LPS·kg^−1^ body weight, applied once 24 h prior to the tissue collection. The injection of appropriate vehicles (phosphate‐buffered saline and/or DMSO) was used as a control. After 72 h following the first injection of R‐BEL or vehicle, the mice were sacrificed as described above, and lung and spleen tissues were excised and frozen in liquid nitrogen. Tissues were homogenized as described, and from portions of homogenates, mitochondria were isolated. Homogenates and isolated mitochondria were estimated for protein carbonyls as described below. For the determination of interleukin‐6 (IL‐6) levels, blood sera were collected 6 h following the LPS treatment. The IL‐6 levels were determined by an enzyme‐linked immunosorbent assay, using commercially available kit reagents (BioVendor, Brno, Czech Republic) according to the manufacturer's instructions.

### Isolation of lung and spleen mitochondria

Typically, 10 wt mice per experiment were sacrificed at 10–15 weeks of age, as were 10 UCP2‐KO gender‐ and sibling‐paired mice. Mice were first stunned and then killed by cervical dislocation. Lungs and spleen were quickly removed and placed separately in ice‐cold isolation medium (180 mm KCl, 5 mm potassium‐MOPS, 5 mm potassium‐EGTA, pH 7.2). Each type of pooled tissue was individually washed twice with isolation medium to remove blood and then minced with scissors while immersed in isolation medium supplemented with BSA (5 mg·mL^−1^). The tissue was homogenized three times with a motor‐driven Potter‐Elvehjem tissue grinder at 0–5 °C. The homogenates were centrifuged at 1000 ***g*** for 5 min at 5 °C, and the pellets were discarded. The supernatants were passed through cheesecloth and centrifuged at 8000 ***g*** for 10 min at 5 °C. The resulting tissue homogenates devoid of nuclei and mitochondria were saved for subsequent protein carbonyl determination. The mitochondria‐containing pellets were suspended in and washed twice with isolation medium and centrifuged between washes at 8000 ***g*** for 10 min at 5 °C. The first wash contained BSA (5 mg·mL^−1^). The final mitochondrial pellets were suspended in isolation medium at a protein concentration of 10 mg·mL^−1^.

### Quantification of protein carbonylation

The reaction between protein carbonyls and 2,4‐dinitrophenylhydrazine (DNPH) or fluorescein‐5‐thiosemicarbazide (FTC) was monitored from excitation spectra measured on a RF‐5301PC spectrofluorometer (Shimadzu, Kyoto, Japan). Intact mitochondria were solubilized at room temperature in 100 mm Na/HEPES buffer, pH 6.0, containing 1% sodium dodecyl sulfate (SDS) and centrifuged at 10 000 ***g*** for 10 min at 4 °C. The absorbance ratio 280/260 of tissue homogenates and solubilized mitochondria was determined to be more than 1 (Olis Cary 14 UV/VIS/NIR spectrophotometer; Olis, Bogart, GA, USA), indicating that there were negligible levels of contaminating nucleic acids. Samples of tissue homogenates or solubilized mitochondria were treated with 0.8 mL of 20 mm DNPH or 20 mm FTC in 2.5 m HCl for 60 min at room temperature in the dark. The reaction was stopped by the addition of 1 mL of 20% trichloroacetic acid (TCA), and the TCA‐treated samples were incubated on ice for 5 min and centrifuged at 10 000__ ***g*** for 10 min at 4 °C. The precipitates were washed three times in 1 mL of 1 : 1 ethanol/ethyl acetate solution and centrifuged as above. The final pellet was dissolved in 0.7 mL of 6 m guanidine hydrochloride and 20 mm KH_2_PO_4_ solution, pH 2.3 (adjusted with HCl). The total carbonyl‐DNPH adducts were detected at the excitation of 346 nm (slit width 20 nm) and emission of 445 nm (slit width 20 nm), and the total carbonyl‐FTC adducts were detected at the excitation of 484 nm (slit width 20 nm) and emission of 521 nm (slit width 20 nm), measured at 25 °C under constant stirring in a 5‐mm quartz cuvette. The fluorescence values were normalized to protein concentration and set relative to the corresponding wt control for each particular experiment.

### Protein oxidation standard curves

Standard curves of protein carbonylation were constructed by mixing varying proportions (0–100%) of oxidized and reduced BSA, following the protocol of Buss *et al*. 23. Briefly, fully reduced BSA was prepared by reacting a 1 g per 100 mL solution in phosphate‐buffered saline (0.14 m sodium chloride, 10 mm sodium phosphate, pH 7.4) with 2 g per 100 mL sodium borohydride for 30 min, followed by neutralizing with HCl. Fully oxidized BSA was prepared by reacting BSA (50 mg·mL^−1^) with hypochlorous acid (5 mm). Albumin solutions were stored at −80 °C. The reaction of DNPH and BSA was carried out as described above. The carbonyl content of a respective BSA standard was determined from A_360_ (Olis), using the molar absorption coefficient of 22 000 m ^−1^·cm^−1^.

### Statistical analysis

Values are expressed as mean ± standard deviation. One‐way ANOVA was used to assess the differences among the groups, and *P* values of <0.05 were considered statistically significant. All calculations were performed using sigmaplot data analysis software (Systat Software Inc., San Jose, CA, USA).

## Results

### Protein carbonyl levels in lung and spleen and mitochondria from wt and UCP2‐KO mice

**Figure 1:**
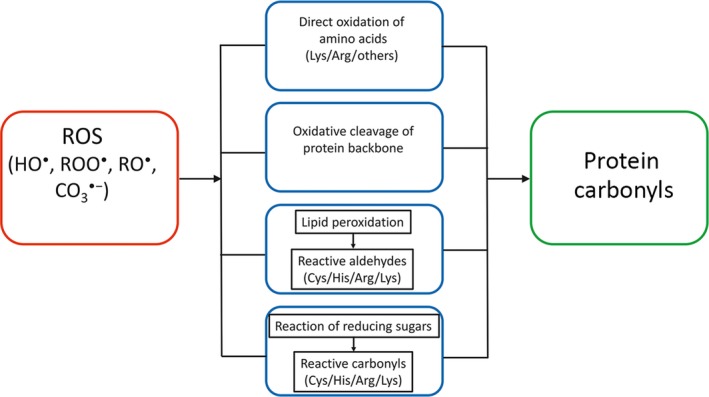
Pathways of oxidant‐induced protein carbonyl formation. The primary superoxide ($O_{2}^{\cdot -}$) is formed by one electron reduction of oxygen by the mitochondrial electron transport chain. Only subsequent oxidants, downstream of hydrogen peroxide (H_2_O_2_), directly oxidize certain amino acid residues or lead to oxidative cleavage of the protein backbone, both resulting in protein carbonylation. These are hydroxyl radical (HO ^·^), peroxyl radical (ROO ^·^), alkoxyl radical (RO ^·^), and carbonate CO_3_^·^^−^ radical 32. Indirect formation of protein carbonyls includes the reaction of cysteine (Cys), histidine (His), arginine (Arg), and lysine (Lys) residues with reactive aldehydes formed by nonenzymatic cleavage of lipid hydroperoxides, and the reaction of reducing sugars or their oxidation products with the same residues (modified from Ref. 22). The majority of protein carbonyls react with DNPH 21.

**Figure 2:**
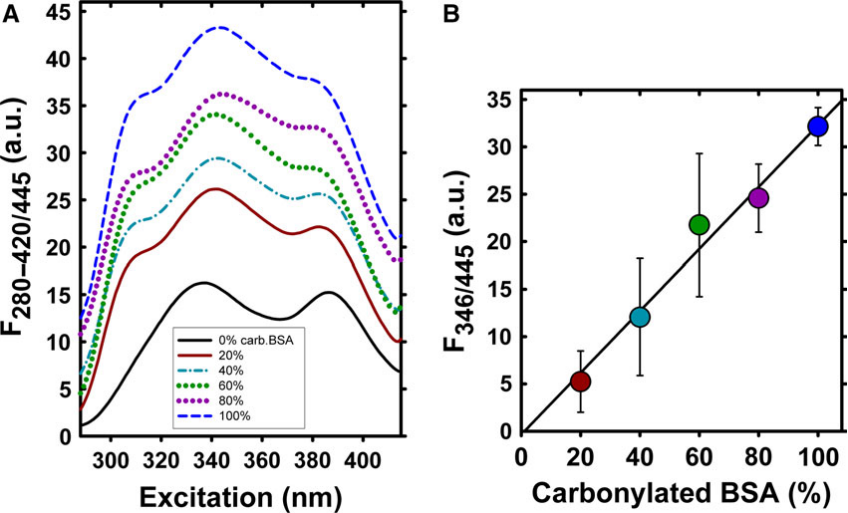
DNPH derivatization of oxidized BSA. DNPH excitation spectra (A) and corresponding standard curves (B) of oxidized BSA. Samples for standard curves were prepared by mixing borohydride‐reduced and HOCl‐oxidized BSA in varying proportions to maintain a constant protein concentration, as described in the ‘Materials and methods’ section. In (B), the fully reduced BSA has been subtracted from the detected DNPH excitation wavelength values.

Protein carbonyls represent an irreversible form of protein oxidation and belong to stable markers of oxidative stress 21, 22. Protein carbonyls are formed early during oxidative stress conditions and are not a result of one specific oxidant, and thus belong to a general marker of overall oxidative stress (Fig. 1). To determine the effect of oxidant‐induced protein carbonylation, we used DNPH derivatization of protein carbonyls and followed the excitation spectra of DNPH fluorescence. The suitability and sensitivity of the method were first tested by mixing varying proportions (0–100%) of oxidized and reduced BSA, as shown in Fig. 2.

**Figure 3:**
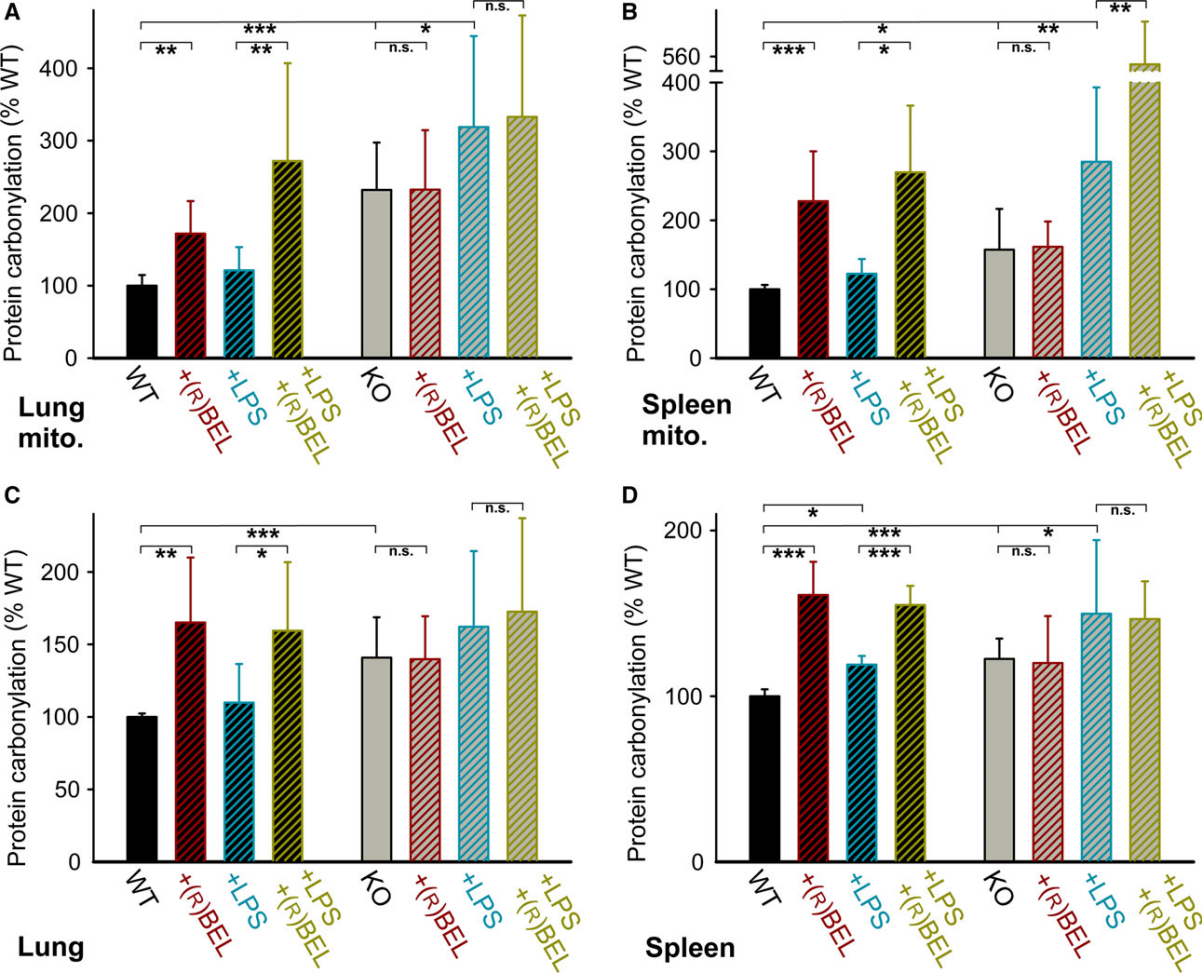
Protein carbonylation in lung and spleen mitochondria and tissue homogenates. Shown are levels of total DNPH adducts, corresponding mainly to protein carbonyl levels, determined from mitochondria isolated from wt mice (black bars) and UCP2‐KO mice (gray bars). The detected DNPH fluorescence values were normalized to protein concentration and set relative to the corresponding wt control for each particular experiment. (A) Lung mitochondria (determined from 10 to 32 estimations), (B) spleen mitochondria (8–16 estimations), (C) lung tissue homogenates (8–19 estimations), and (D) spleen tissue homogenates (7–13 estimations). Where indicated, mice (10–15 weeks of age) were injected subcutaneously with a selective inhibitor of iPLA2γ, R‐BEL (1 mg·kg^−1^ body weight), or intraperitoneally with LPS (5 mg·kg^−1^ body weight) or both. One‐way ANOVA: *P <* *0.05; **P <* *0.02; ***P <* *0.001.

To test the hypothesis of UCP2‐dependent antioxidant mechanism, we measured DNPH adducts, corresponding mainly to protein carbonyl levels, in mitochondria isolated from selected tissues of wt and UCP2‐KO mice. The total levels of DNPH adducts were normalized to the protein concentration and set relative to the wt control. In a typical experiment, the determined baseline protein carbonyl levels of wt controls were around 2 nmol·mg^−1^ protein (not shown). As shown in Fig. 3, the baseline protein carbonyl levels in lung and spleen mitochondria from the UCP2‐KO mice were by ~ 2.3‐fold and ~ 1.5‐fold higher, respectively, when compared to age‐matched wt mice (Fig. 3A,B; bars WT vs. KO). A subcutaneous injection of a selective iPLA2γ inhibitor R‐BEL, applied in three doses 24 h apart for total of 72 h, led to an about twofold increase in the DNPH adducts in wt animals, consistent with an iPLA2γ‐dependent increase in mitochondrial steady‐state protein carbonyl levels (Fig. 3A,B; bars WT vs. WT+R‐BEL). In contrast, identical R‐BEL treatment of UCP2‐KO animals caused no further increase in the DNPH adducts compared to the control (Fig. 3A,B; bars KO vs. KO+R‐BEL), indicating that the pharmacological inhibition of iPLA2γ causes no further increase in the acute oxidative stress when UCP2 is absent.

The endotoxin LPS is a powerful tool to investigate the response to acute oxidative stress and ROS signaling 24, 25. An intraperitoneal injection of LPS 24 h prior to the tissue collection did not lead to significant changes in the steady‐state protein carbonyl levels detected in lung and spleen mitochondrial isolated from wt mice (Fig. 3A,B; bars WT vs. WT+LPS), but led to an increase in the detected carbonyls in both the lung and spleen mitochondria isolated from the UCP2‐KO mice (Fig. 3A,B; bars KO vs. KO+LPS). Finally, the injection of both R‐BEL and LPS has markedly elevated the mitochondrial steady‐state protein carbonyl levels in wt mice (Fig. 3A,B; bars WT+LPS vs. WT+LPS+R‐BEL), whereas no further effect of R‐BEL in addition to LPS was observed in lung mitochondria of UCP2‐KO mice (Fig. 3A; bars KO+LPS vs. KO+LPS+R‐BEL). These results are consistent with the relevant antioxidant mechanism being dependent on both UCP2 and iPLA2γ. For reasons unidentified to date, combined administration of R‐BEL plus LPS resulted in higher levels of total DNPH adducts in spleen mitochondria (Fig. 3B; bars KO+LPS vs. KO+LPS+R‐BEL). Qualitatively similar data were observed, when we detected protein oxidation using the protein carbonyl‐selective fluorescent probe FTC (data not shown).

### Protein carbonylation levels in lung and spleen tissues from wt and UCP2‐KO mice

We aimed to investigate whether the expected UCP2‐mediated antioxidant protection can be manifested also within the cell cytosol. Thus, we have quantified the protein carbonyl levels in tissue homogenates from which mitochondria were removed by differential centrifugation. As shown in Fig. 3C,D, the tissue homogenates exhibited a pattern similar to the one obtained with isolated mitochondria.

Thus, the tissue homogenate DNPH adducts, corresponding to protein carbonyl levels, were ~ 1.4‐fold and ~ 1.2‐fold higher in lung and spleen samples, respectively, of UCP2‐KO mice compared to the wt mice (Fig. 3C,D; bars WT vs. KO). In addition, the tissue homogenates obtained from the wt mice showed ~ 1.6‐fold increase in the protein carbonyl levels following the R‐BEL challenge for both lung and spleen (Fig. 3C,D; bars WT vs. WT+R‐BEL). Similar to the results observed with isolated mitochondria, the R‐BEL challenge did not increase tissue homogenate protein carbonyl levels in lung and spleen samples of UCP2‐KO mice (Fig. 3C,D; bars KO vs. KO+R‐BEL). LPS administration did not increase tissue homogenate protein carbonyl levels in lung and spleen samples of wt mice (Fig. 3C,D; bars WT vs. WT+LPS), but caused increases in the protein carbonyl levels in lung and spleen of UCP2‐KO mice (Fig. 3C,D; bars KO vs. KO+LPS). No further effect of R‐BEL in addition to LPS was observed in lung and spleen tissue homogenates of UCP2‐KO mice (Fig. 3C,D; bars KO+LPS vs. KO+LPS+R‐BEL). Taken together, these results indicate that the iPLA2γ‐regulated UCP2 antioxidant mechanism can manifest its function also in the cell cytosol of the corresponding tissue.

### Suppression of the inflammatory response by UCP2 correlates with the activity of mt‐iPLA_2_γ

**Figure 4:**
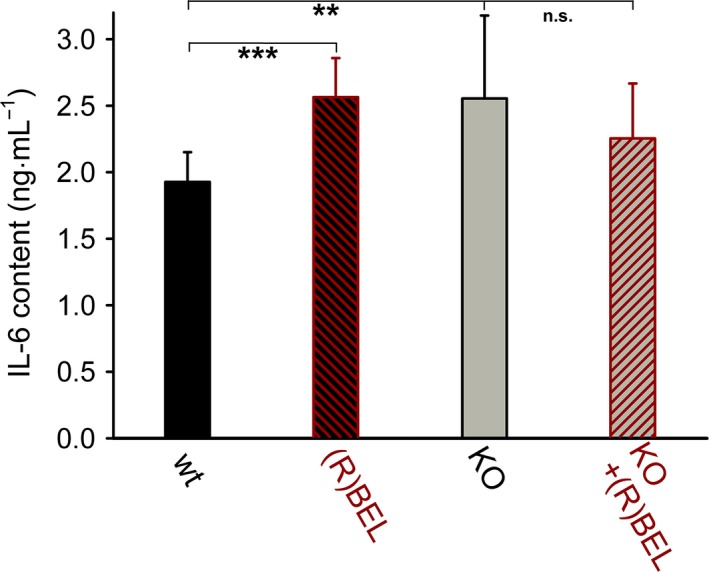
IL‐6 levels after the inflammatory response in lung from wt and UCP2‐KO mice. The lung inflammatory response induced by 5 mg of lipopolysaccharide·kg^−1^ body weight is expressed as the serum levels of IL‐6. The serum was collected 6 h following the LPS injection. Where indicated, mice (10–15 weeks of age) were injected subcutaneously with a selective inhibitor of iPLA2γ, R‐BEL (1 mg·kg^−1^ body weight). One‐way ANOVA: **P <* *0.02; ***P <* *0.001.

Lipopolysaccharide injection has been widely used to study acute inflammatory response, associated with increased plasma levels of *pro‐*inflammatory cytokines 26, and LPS administration is known to cause a dose‐dependent increase in IL‐6 concentration 26. Therefore, the *in vivo* relevance of the indicated iPLA2γ‐dependent UCP2 antioxidant mechanism was further tested by investigation of the increase in the *pro*‐inflammatory cytokine IL‐6 upon the LPS‐induced challenge. The determined baseline serum levels of IL‐6 ranged between 10 and 20 ng·mL^−1^ (not shown). Figure 4 shows that the IL‐6 levels in mouse sera collected 6 h following the LPS injection increased to about 1200 ng·mL^−1^ and were ~ 2.1‐fold higher in UCP2‐KO mice compared to the age‐matched wt controls. A subcutaneous injection of a selective iPLA2γ inhibitor R‐BEL, applied in three doses 24 h prior to the addition of LPS, led to a further increase in serum IL‐6 levels in wt mice. In contrast, R‐BEL caused no further increase in serum IL‐6 levels in UCP2‐KO mice (Fig. 4; +R‐BEL). These data demonstrate that the *pro*‐inflammatory effect of LPS is further amplified by UCP2 ablation, and pharmacological inhibition of iPLA2γ does not lead to an additional LPS‐induced increase in IL‐6 serum levels in the absence of UCP2.

## Discussion

In this work, we demonstrate the effect of acute *pro*‐oxidant and *pro*‐inflammatory conditions, while simultaneously providing challenges, which either block the function of mitochondrial uncoupling protein UCP2 (studying tissues of the UCP2‐knockout mice) or inhibit the function of mitochondrial calcium‐independent phospholipase A2γ (mt‐iPLA2γ). We show that the steady‐state protein carbonyl levels were found higher in lung and spleen mitochondria and tissues of UCP2 KO mice, suggesting UCP2 as a key element promoting the antioxidative processes preventing nonenzymatic protein oxidation. As the UCP2‐related antioxidant protection can be abolished by the selective mt‐iPLA2γ inhibitor R‐BEL, the revealed data support the hypothesis of an antioxidant synergy of UCP2 and mt‐iPLA_2_γ *in vivo*. This synergy is revealed to be a significant cytoprotective mechanism in lung and spleen.

**Figure 5:**
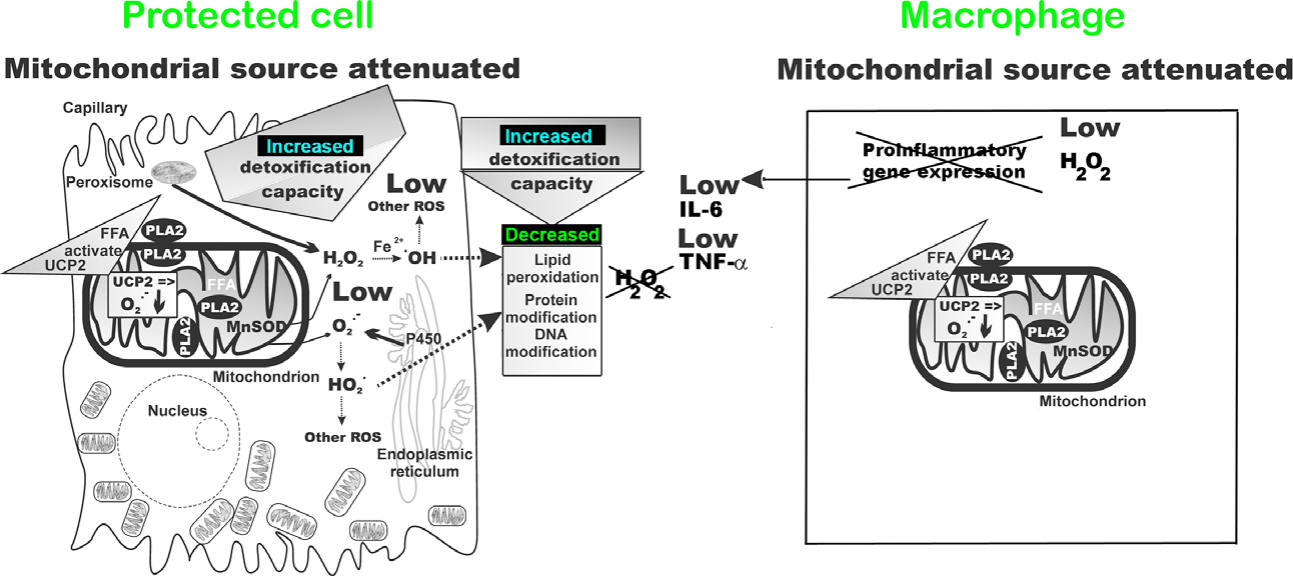
Schematics for ROS homeostasis, at the cellular and mitochondrial levels, by concerted action of mt‐iPLA_2_γ and UCP2. Feedback downregulation of cellular oxidative stress. Oxidative stress and/or redox regulation in the cytosol induce mt‐PLA_2_‐mediated cleavage of free fatty acids (FFA) that initiate UCP2‐mediated uncoupling. Uncoupling decreases the production of mitochondrial superoxide ($O_{2}^{\cdot -}$) and subsequent H_2_O_2_, produced by the mitochondrial matrix manganese superoxide dismutase (MnSOD). Consequently, release of H_2_O_2_ and superoxide to the cytosol decreases, which leads to an increase in the cellular antioxidant capacity that had previously been used to reduce the mitochondrial oxidants. The increased reductive and detoxification capacity can then be utilized to shift the overall redox balance of the cell, resulting in decreased lipid peroxidation and protein and DNA oxidation.

We can interpret the observed increases in protein carbonylation in lung and spleen tissues and mitochondria from UCP2‐KO mice and upon R‐BEL administration to wt mice as originating from the absence of a protective mechanism *in vivo*. We suggest that this protective mechanism arises from the concerted action of mt‐iPLA_2_γ and UCP2 and may increase survival rates for diseases originating from oxidative stress and from inflammation. We interpret our results in terms of a blockage of the mt‐iPLA_2_γ‐ and UCP2‐mediated antioxidant effect after either R‐BEL inhibition or UCP2 ablation. Our previous reports have shown that H_2_O_2_ activates mt‐iPLA2γ, resulting in an instant UCP2‐dependent suppression of mitochondrial superoxide formation in isolated lung and spleen mitochondria 10 and pancreatic β‐cells 11. The inability to activate this mechanism in UCP2‐KO mice has been reflected by the increased protein carbonyl levels (as the oxidative stress marker) in mitochondria and tissue homogenates of lung and spleen tissues. The described synergy provides a cytoprotective function by a feedback downregulation of mitochondrial superoxide formation (Fig. 5).

Our interpretation of mt‐iPLA2γ participation in the regulation of UCP2 is based solely on the effect of a selective pharmacological iPLA2γ inhibitor, R‐BEL. The discovery of this R‐enantiomer of bromoenol lactone and its high selectivity for iPLA2γ provided a long‐needed pharmacological tool to selectively inhibit iPLA2γ without blocking the function of other phospholipases 27. However, haloenol lactones have been first identified as chymotrypsin inhibitors 28 and racemic bromoenol lactone has been shown to inhibit phosphatidate phosphatase and other esterases 29, which may provide other potential cellular targets of R‐BEL and introduces limitations when using R‐BEL in complex cellular systems. Nevertheless, none of the other potential targets of R‐BEL is expected to directly regulate the function of UCP2 in mitochondria. Therefore, we deduce that the correlation between the effects of R‐BEL and ablation of UCP2 is consistent with the outlined hypothesis of mt‐iPLA2γ‐dependent antioxidant activity of UCP2.

The mechanism that we have outlined might counteract oxidative stress arising from a variety of origins. When the initial oxidative stress is of cytosolic origin (e.g., activation of NADPH oxidase or reactions involving cytochrome P_450_), diminished mitochondrial production of superoxide (leading to diminished downstream ROS production, such as H_2_O_2_ production) would allow antioxidant mechanisms that suddenly acquired a greater capacity, which had been previously used for scavenging of mitochondria‐released ROS, to deal with the excessive external oxidative stress 3, 4, and hence would attenuate the external oxidative stress (Fig. 5). This stress might even be a pro‐inflammatory response. When the oxidative stress is mitochondrial in origin, our suggested mechanism is consistent with a direct feedback attenuation of mitochondrial ROS production.

A concerted action of mt‐iPLA_2_γ and UCP2 might interfere with (or regulate) other cellular redox regulation pathways. We have brought an example, where redox regulations of IL‐6 levels upon inflammatory response to LPS are most probably tuned to moderate levels by an antioxidant concerted action of mt‐iPLA_2_γ and UCP2, as evidenced by elevation of IL‐6 levels under conditions when the concerted action is blocked on mt‐iPLA_2_γ side by R‐BEL. Although mt‐iPLA2γ is present in UCP2‐KO mice, R‐BEL does not cause further increase in serum IL‐6 levels, consistent with the function of iPLA2γ being coupled to the antioxidant function of UCP2. We may speculate that redox regulations of the cytokine expression are amplified by the pro‐oxidant role of R‐BEL, whereas the antioxidant function of UCP2 in concert with mt‐iPLA2γ permits lower IL‐6 levels.

The significance of our suggested feedback downregulation of oxidative stress by the concerted action of UCP2 and mt‐iPLA_2_γ in lung and spleen tissues can be assessed for the various tissue types of the lung. Lung tissue is composed of over 40 different cell types with variable mitochondrial densities and roles 30. Bronchial branches, accompanied by branches of the pulmonary artery, nerves, and lymph vessels, usually first pass through the intersegmental and interlobular sheets of connective tissue. Bronchi also contain glands, several small pieces of supporting cartilage, and a layer of smooth muscle located between the cartilage and epithelium. The epithelium usually represents a much smaller mass than does the underlying connective tissue. In turn, the epithelium of the alveoli is formed by two cell types, alveolar type I cells, which are extremely flat cells (~ 0.05 μm in depth) of complex shape that form up to 95% of the alveolar wall surface; and alveolar type II cells, which have irregular (even cuboidal) shapes, form small bulges on the alveolar walls, and contain a large number of granules containing pulmonary surfactant (the mixture of phospholipids that maintains a low surface tension in alveoli). Alveolar macrophages migrate freely over the alveolar epithelium and ingest particulate matter. If all the aforementioned cells contain UCP2 and mt‐iPLA_2_γ, the described feedback downregulation of oxidative stress may contribute substantially to their redox equilibria.

The spleen also has a complex histology 31. The principal contribution of the red and white pulp parenchyma tissues as well as lymphocytes or macrophages to the mitochondrial population can be assumed. Because UCP2 is abundant in immune cells, defense against oxidative stress in the complex compartments of spleen would be beneficial.

In conclusion, the presented data extend our previous findings showing antioxidant activity by a synergy of redox‐sensitive mt‐iPLA2γ and UCP2 in lung and spleen 10. By determining the relative levels of protein carbonyls in isolated lung and spleen mitochondria and lung and spleen tissue, together with determining the levels of IL‐6 in serum, we show that acute *pro*‐oxidative and *pro*‐inflammatory conditions yield higher levels of protein oxidation and higher levels of IL‐6 in the absence of UCP2 or in the presence of selective inhibitor of iPLA2γ. Thus, these results provide additional support of the antioxidant and anti‐inflammatory action of UCP2 by a mechanism involving mitochondrial iPLA2γ.

## Author contributions

JJ and MJ conducted measurements and calculated data, and MJ and PJ designed experiments, analyzed the data, and wrote the manuscript.
